# Investigating the Roles of the C-Terminal Domain of *Plasmodium falciparum* GyrA

**DOI:** 10.1371/journal.pone.0142313

**Published:** 2015-11-13

**Authors:** Soshichiro Nagano, Eiko Seki, Ting-Yu Lin, Mikako Shirouzu, Shigeyuki Yokoyama, Jonathan G. Heddle

**Affiliations:** 1 Heddle Initiative Research Unit, RIKEN, 2–1 Hirosawa, Wako-shi, Saitama, 351–0198, Japan; 2 RIKEN Structural Biology Laboratory, 1-7-22 Suehiro-cho, Tsurumi-ku, Yokohama, 230–0045, Japan; 3 Division of Structural and Synthetic Biology, RIKEN Center for Life Science Technologies, 1-7-22 Suehiro-cho, Tsurumi-ku, Yokohama, 230–0045, Japan; Université Pierre et Marie Curie, FRANCE

## Abstract

Malaria remains as one of the most deadly diseases in developing countries. The *Plasmodium* causative agents of human malaria such as *Plasmodium falciparum* possess an organelle, the apicoplast, which is the result of secondary endosymbiosis and retains its own circular DNA. A type II topoisomerase, DNA gyrase, is present in the apicoplast. In prokaryotes this enzyme is a proven, effective target for antibacterial agents, and its discovery in *P*. *falciparum* opens up the prospect of exploiting it as a drug target. Basic characterisation of *P*. *falciparum* gyrase is important because there are significant sequence differences between it and the prokaryotic enzyme. However, it has proved difficult to obtain soluble protein. Here we have predicted a new domain boundary in *P*. *falciparum* GyrA that corresponds to the C-terminal domain of prokaryotic GyrA and successfully purified it in a soluble form. Biochemical analyses revealed many similarities between the C-terminal domains of GyrA from *E*. *coli* and *P*. *falciparum*, suggesting that despite its considerably larger size, the malarial protein carries out a similar DNA wrapping function. Removal of a unique Asn-rich region in the *P*. *falciparum* protein did not result in a significant change, suggesting it is dispensable for DNA wrapping.

## Introduction

**Fig 1:**
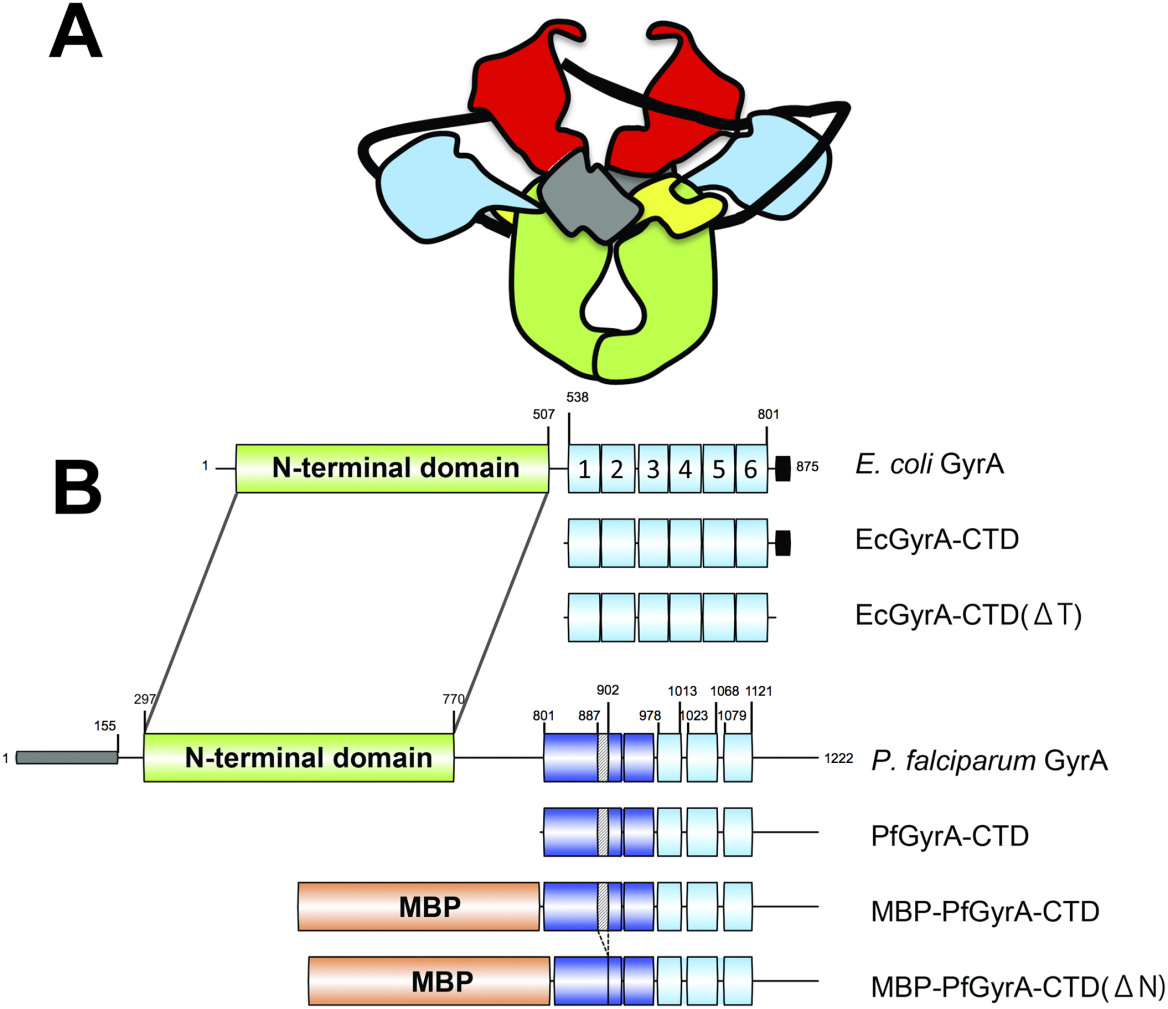
DNA gyrase structure. **A** Schematic of DNA gyrase bound to DNA prior to ATP binding. DNA is shown in black. The N-terminus of GyrB is shown in red and the C-terminus in grey and yellow. The N and C-termini of GyrA are shown in green and blue respectively. **B** Schematic of domains in gyrase proteins and constructs used in the present study. β-pinwheel blades are numbered for *E*. *coli* GyrA. Colouring scheme is as follows; N-terminal domain, green; MBP, orange; β-pinwheel motifs identified by the Pfam server [5], light-blue; putative β-pinwheel motifs, dark-blue; signal/transit peptide, grey; acidic C-terminal tail, black. Segments with diagonal fill indicate the Asn-rich region (residues 887–902). Numbers refer to amino acid positions (see text).

DNA gyrase is a type II topoisomerase (type II topo) with the unique capability of introducing negative supercoils into DNA (Fig 1A). It consists of two proteins, GyrA and GyrB, which form an A_2_B_2_ complex in the functioning enzyme whose arrangement and overall structure is known at low resolution [1]. Gyrase is found in prokaryotes and some lower eukaryotes and it is often accompanied by topoisomerase IV (topo IV) a second type II topo that possesses complementary activity, showing a preference for decatenating topologically linked DNAs and relaxing supercoiled DNA [2]. Like gyrase, topo IV is a heterotetramer with ParC and ParE proteins being the equivalent of GyrA and GyrB respectively. Topo IV can relax both positively and negatively supercoiled DNA, but the former is the preferred substrate. Despite the complementary activities of gyrase and topo IV, some organisms possess gyrase as the sole type II topo (for example *Mycobacterium tuberculosis* [3, 4]).

It is known that the C-terminal domains (CTD) of GyrA and ParC play major roles in substrate specificities of the holoenzymes and seem to account for the differences in activities between the two [6]. The CTD is a β-pinwheel structure [7] made up of repeating “blade” motifs. In GyrA, this β-pinwheel domain is involved in wrapping substrate DNA to give the specific positive wrap necessary to ensure the correct “handedness” of negative supercoiling [6] (Fig 1A). Topo IV’s inability to negatively supercoil DNA is due to differences in the CTD compared to gyrase. The key difference is in the loop region of the first β-pinwheel blade: A motif with the consensus sequence of QXXGGXG, termed the GyrA-box [8] is present in GyrA and it has been shown that abrogation of this motif leads to the loss of the ability of gyrase to supercoil DNA [9] and to bend DNA when CTD is presented as an isolated fragment [10]. Additionally, degenerate versions of GyrA-box motifs found in the loop regions of blades other than the first blade also play roles: Mutating the glycines of “GyrA-box-l” in the fifth blade of *M*. *tuberculosis* GyrA leads to loss of DNA supercoiling and decatenation activities [11], and mutating the arginine residues of the degenerate GyrA-boxes in the CTD of *E*. *coli* ParC leads to changes in its processivity and/or rate of reaction [12].

Genes encoding gyrase have been discovered in the genome of *Plasmodium falciparum*, the causative agent of the most destructive form of malaria [13] and the enzymes are localised to the apicoplast, a relict plastid [14]. The presence of an enzyme long thought to be exclusive to prokaryotes in *P*. *falciparum* can be explained by the apicoplast’s complex evolutionary history: The apicoplast evolved by two stages of endosymbiosis, the first stage being the origin of algae in which photosynthetic cyanobacteria were engulfed and became the chloroplast of the host. A second endosymbiosis occurred when the algal cell was itself engulfed by the precursor of *Plasmodium*. The engulfed algae gave rise to the apicoplast, an indispensable organelle, which is involved in synthesis of compounds such as fatty acids, isoprenoids, and iron-sulphur clusters [14]. The apicoplast is surrounded by three or four membranes, reflecting its unusual origin [14].

Prokaryotic gyrase is well-characterised and is a proven target for antimicrobial drugs [15] due to the fact that its catalytic mechanism involves the temporary formation of double strand breaks in the substrate DNA, breaks which can be trapped by some drugs, leading to a lethal fragmentation of the genome. The discovery of a prokaryotic gyrase-like protein in *Plasmodium* presents a possible new target for either existing gyrase-targeting antibiotics or, given the proven effectiveness of gyrase as a lethal target, for development of novel therapeutics against the *Plasmodium* enzyme [16]. Indeed, a number of pieces of research provide experimental evidence for (fluoro)quinolone targeting of apicoplast gyrases [17–22]. From an evolutionary standpoint, *Plasmodium* gyrase is interesting as it exhibits a divergence from prokaryotic counterparts [23] and also because it is the sole type II topo in the apicoplast [24], raising questions over how regulation of DNA topology is achieved. It is noteworthy that drug-resistant malaria is a growing problem and the estimated number of cases of malaria reached 207 million in 2013 [25]. This could increase in the future including in regions outside those where malaria is currently endemic, as a widening of the habitable zones of the *Anopheles* mosquito vector occurs due to global warming.

In the present study, the boundary of the C-terminal domain of GyrA of *P*. *falciparum* (PfGyrA-CTD) was predicted using a bioinformatics approach. Additionally, the coding sequence of the C-terminal fragment was subsequently cloned, expressed, purified, and subjected to biochemical analyses along with the control protein; the C-terminal domain of *E*. *coli* GyrA (EcGyrA-CTD). Furthermore, a deletion mutant of PfGyrA-CTD was created in order to probe the role of an Asn-rich region unique to the *P*. *falciparum* protein, and collectively the present study aims to deepen our understanding of the mechanism of *P*. *falciparum* gyrase as part of a long term goal to fully understand its structure and function so that new anti-malarial therapeutics may be developed that target it.

## Material and Methods

### Bioinformatics analyses

Sequence alignment was carried out with ClustalW 2.1 [26] using default settings, and aligned sequences were viewed with CLC sequence viewer 6.8.1 (CLC bio). Domains were predicted from protein primary structures using the Pfam [5] and SMART [27] servers.

### Cloning, expression, and protein purification

For a schematic of domains in gyrase proteins and constructs produced, see Fig 1B. All primers used in the present study were purchased from Eurofins, and are listed in the Supporting Information. Sequences of all cloned DNAs were verified by the Sanger dideoxy termination method [28]. Synthetic DNA equating to the coding sequence of *P*. *falciparum* GyrA residues 156–1222 which has been codon optimised for overproduction in *E*. *coli* was purchased from Genscript. The coding sequence of PfGyrA-CTD (residues 801–1222) was amplified by PCR, and cloned into pCR2.1, a plasmid for cell-free protein synthesis using the In-Fusion^®^ HD Cloning kit (Takara). The construct of PfGyrA-CTD was further modified by inserting the DNA sequence GGCAGC (encoding Gly-Ser) between the coding regions of the TEV cleavage site and PfGyrA-CTD. Insertion of Gly-Ser residues was carried out in order to improve the cleavage of the N-terminal His tag with TEV protease.

Coupled transcription–translation cell-free protein synthesis was performed as described previously [29] with minor modifications. First, polyethylene glycol (PEG, average MW 8,000), which enhances protein aggregation and precipitation, was not used in the internal and external solutions. For the cell-free protein synthesis with chaperons, S30 extract prepared from *E*. *coli* cells overproducing the early chaperons (DnaK, DnaJ and GrpE) was used. Cell-free protein synthesis reactions were performed in dialysis mode at 25°C overnight. All the constructs were expressed with N-terminal His-tags. Synthesized proteins were centrifuged, supernatant recovered and affinity purified with a His-trap column or a Ni Sepharose 6 fast flow column (GE Healthcare). TEV protease cleavage was carried out at 4°C overnight, dialysing against 20 mM Tris-HCl buffer (pH 8.0) containing 300 mM NaCl and 1 mM DTT. The proteins were loaded onto a cation exchange column HiTrap SP HP (GE Healthcare) and binding fractions eluting at approx. 1 M NaCl were collected. The purified proteins were stored at -80°C in 20 mM Tris-HCl buffer (pH 8.0) containing 300 mM NaCl, 1 mM DTT and 20% glycerol. PfGyrA-CTD with and without a N-terminal His-tag will be referred to as His-PfGyrA-CTD and PfGyrA-CTD, respectively. Additionally, the coding sequence of PfGyrA-CTD was cloned into pMALX(E) [30] using the NotI and BamHI sites to produce a chimeric PfGyrA-CTD bearing a N-terminal maltose binding protein (MBP-PfGyrA-CTD). The chimeric protein was overproduced in BL21(DE3) cells (Novagen), and then affinity purified using amylose resin (New England Biolabs), followed by size exclusion chromatography (HiPrep 16/60 S-200 HR, GE Healthcare) using a high-salt buffer (1 M NaCl, 5 mM D-Maltose, 20 mM Tris-HCl, pH 8.0) to separate the protein from DNA. Typical flow rate was 0.4 ml/min, and the sample volume was 4 ml.

The coding sequence of *E*. *coli* GyrA CTD (corresponding to residues 531–875, EcGyrA-CTD) was cloned into pET28(a)+ (Novagen) using the NdeI and XhoI sites. A truncated (residues 531–853) construct of EcGyrA-CTD without the C-terminal acidic tail region was produced by changing the codon for residue Ser854 (2560-AGT-2562) into a stop codon (TAA). Both versions of EcGyrA-CTD were overproduced and purified similarly to the previously reported protocol [31], except that cleavage of the N-terminal His-tag was performed using thrombin (Nacalai). EcGyrA-CTD with or without the C-terminal acidic tail (residues 854–875) will be distinguished by the suffixes of (WT) and (ΔT), respectively.

### Analytical size exclusion chromatography

Analytical size exclusion chromatography was performed using a Superdex 200 5/150 GL column (GE Healthcare). 10 μl of each protein sample was subjected to isocratic elution with a flow rate of 0.5 ml/min, using buffer containing 300 mM NaCl, 20 mM Tris/HCl, pH 8.0. For MBP fusion proteins, the buffer was supplemented with 5 mM maltose in order to minimise the non-specific interaction between MBP and the resin matrix.

### CD spectroscopy

Circular dichroism of proteins (0.1 mg/ml in CD buffer: 10 mM Na phosphate buffer, pH 8.0) as a function of wavelength (190–350 nm, 1 nm interval) was measured using a J-720 machine (Jasco). Five independent measurements were averaged and smoothed using Means-Movement with a convolution width of 11 (Spectra Analysis, JASCO). The spectrum of the buffer solution was subtracted prior to the deconvolution of spectra using the Dichroweb server [32] employing the CDSSTR program and reference set 4.

### Limited proteolysis

Proteins were subjected to limited proteolysis using trypsin (Nacalai) by a protocol adapted from Noack & Lamparter [33]. 50 μl of the CTD proteins at 2 mg/ml were mixed with 50 μl of trypsin solution (all buffer solutions were 20 mM Tris-HCl, pH 7.9, 300 mM KCl, 10% glycerol, 2 mM β-mercaptoethanol). The reaction mixtures were incubated at 18°C for 1 hour, during which aliquots containing 10 μg of the protein were taken at time intervals of 0, 5, 10, 20, 30, and 60 minutes and added to 10 μl SDS-loading solution to stop the reaction. Proteolysis as a function of time was visualised by SDS-PAGE using 12% gels. Protein bands were excised from the gels and subjected to further trypsinolysis prior to MALDI-TOF analyses. Alternatively, the proteins were blotted onto PVDF membranes using a Trans-Blot Turbo (Biorad) prior to amino acid sequencing with Edman degradation for determining the trypsin cleavage sites.

### DNA binding assay

Binding of DNA by proteins was assayed using the previously reported protocol [34]. The substrate 140 bp linear dsDNA was amplified from pBR322 by PCR using a pair of Cy3-labeled primers. 1 nM of DNA substrate was incubated with proteins at molar excess of between 125–1000 fold in 10 μl reactions. (Reaction buffer: 20 mM Tris-HCl, pH 7.5, 300 mM KCl, 4 mM MgCl_2_, 5 mM DTT, 10% glycerol). The samples were subjected to non-denaturing PAGE using a 5% gel and TBM buffer (98 mM Tris-base, 98 mM boric acid, 4 mM MgCl_2_, pH 8.3) and visualised using a Typhoon 9400 (GE Healthcare). Fluorescence anisotropy was carried out using black 96-well polypropylene plates (Nunc) and a Mithras LB940 multimode microplate reader (Berthold). 37-bp substrate dsDNA was FAM-labelled, and the produced in the same way as previously reported [31]. Protein concentrations ranging between 0–3200 nM were tested while the DNA concentration was held constant at 50 nM. The experiment was carried out using a buffer containing 70 mM KCl, 20 mM Tris-HCl pH 7.5, 10% (v/v) glycerol, and 1 mM MgCl_2_. Curves with the following equation were fitted to experimental data using Kaleidagraph 4.1 (Synergy Software) to model a single ligand binding; *y* = (*Bmax* × *x*) ÷ (*Kd* + *x*). Where *y* and *x* represent the concentrations of DNA and proteins in nM, respectively. *Bmax* and *Kd* represent the maximum fluorescence anisotropy in mA and the equilibrium binding constant in nM, respectively.

### Topology footprinting assay

The ability of C-terminal fragments of GyrA to introduce writhe into DNA was tested in a similar way to that previously reported [31]. In order to emulate the DNA substrate pSG483 described therein, a single recognition site of Nb.BbvCI nicking endonuclease was introduced into pUC19 plasmid (New England Biolabs) using the Quikchange^®^ mutagenesis protocol (Agilent). The plasmid was treated by Nb.BbvCI nicking endonuclease (New England Biolabs) to create a topologically free, relaxed DNA (S1 Fig). Proteins at various molar excesses over the nicked plasmid (300 ng, 0.08 pmol) were incubated at 37°C for 30 minutes in 30 μl reactions. The nick was sealed using 60 units of T4 DNA ligase (New England Biolabs) at 37°C for 1 hour. The protein bound to the DNA was removed by adding EDTA (10 mM final concentration), SDS (1% final concentration) and proteinase K (50 μg/ml final concentration) prior to subjecting the samples to gel electrophoresis using 1% agarose gel and 1X TAE buffer for 1 hour.

## Results

### Bioinformatic analysis and protein production of PfGyrA-CTD

Gyrase proteins from *Plasmodium* spp. have attracted attention for their unusual evolutionary trajectory as well as medical implications as a possible therapeutic target. Successful purification of these proteins will enable their characterisation, and a number of attempts have been made in recent years. Successes include the B-subunit of gyrase (GyrB) from *P*. *vivax* [24], and GyrB from *P*. *falciparum* [35] which has been successfully expressed as the predicted mature form without the signal/transit peptides (residues PvGyrB 106–992, PfGyrB 121–1006). *Plasmodium* GyrA has proved much more difficult, having never been produced as a soluble protein in its mature form. Only truncated fragments have been expressed and purified (PfGyrA 163–540 and 723–887) [35] which does not allow reconstitution of the fully functioning enzyme. In the present study, we initially attempted to produce the mature form of full-length PfGyrA. As this had previously not proved possible, we tried to optimise expression by using a cell-free expression system [29] and a gene sequence optimised for expression in *E*. *coli*. A number of constructs were generated where variations included the residue range (either 156–1222 or 163–1222 [35]), the type of the N-terminal tag (His-tag, TrxA-tag, or SUMO-tag), together with optimisation in expression strategies including variations in the reaction temperature, concentrations of the template DNA, presence of Zn^2+^ or chaperones (DnaK, DnaJ and GrpE), and co-expression or supplementing the synthesis reaction with PfGyrB (residues 121–1006). It is clear that use of the cell-free system was advantageous, with all attempts resulting in expression of full length PfGyrA protein, but insufficient amounts of the soluble protein could be obtained (S2 Fig). Due to these initial difficulties in producing soluble, mature PfGyrA we chose to produce PfGyrA fragments. As these had not yet been identified/purified, bioinformatics approaches were employed to determine the boundary of the C-terminal domain to generate a fragment suitable for biochemical analyses.

As the first step, sequences of *Plasmodium* and prokaryotic gyrases were aligned. Based on the alignment, PfGyrA Asn801 was deemed the suitable starting residue of the PfGyrA-CTD construct, because it is the equivalent of EcGyrA Thr535, the first residue of the CTD construct of EcGyrA used for its structural determination (PDB code 1ZI0 [36]). Subsequently, residues 801–1222, containing the CTD of PfGyrA, were used as a basis for construct design throughout the present study (Fig 1B).

**Fig 2:**
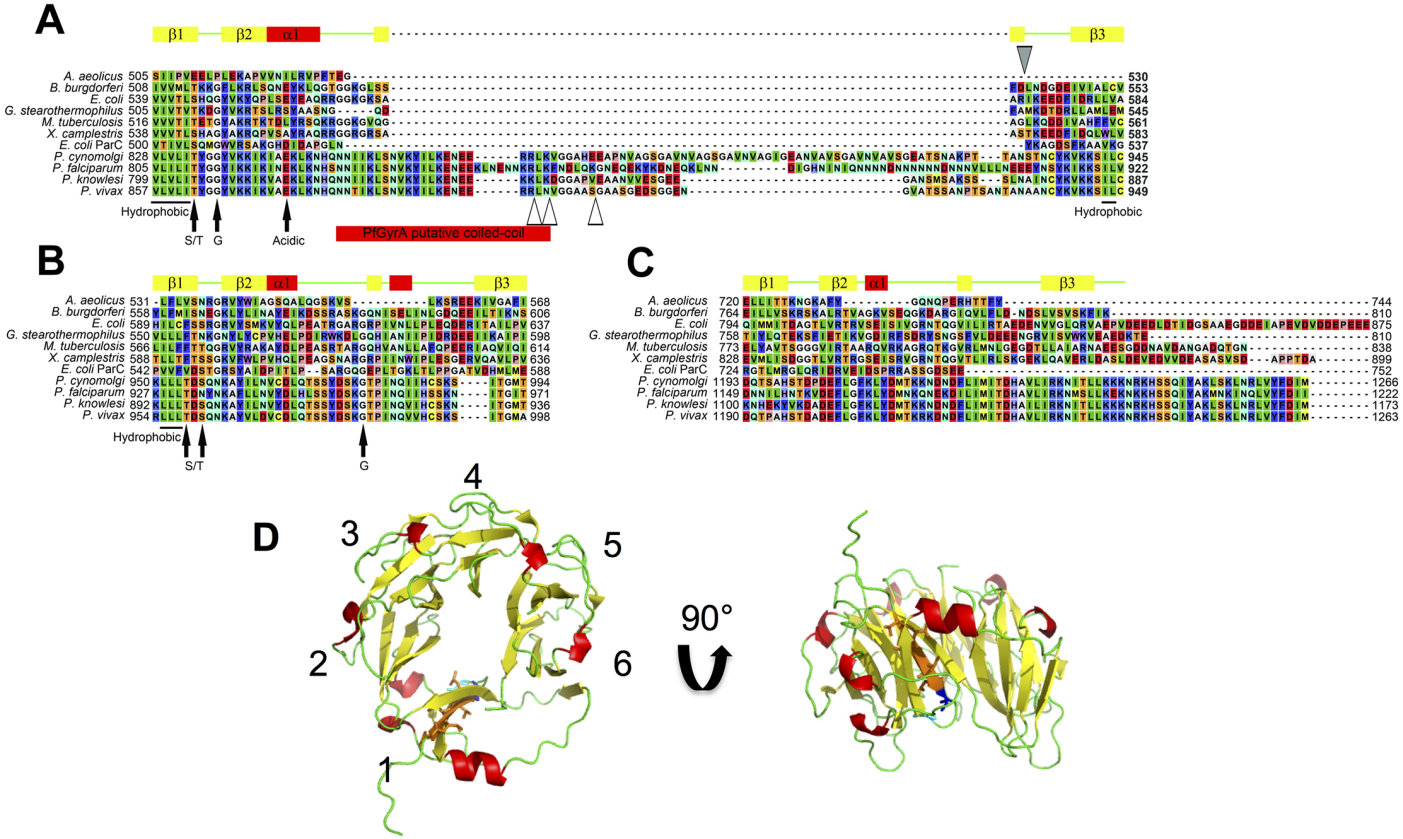
Gyrase β-pinwheel blade motif alignments. Aligned sequences of the region corresponding to the first (**A**), the second (**B**) and the sixth (**C**) β-pinwheel blade motifs of prokaryotic gyrases. Red, yellow and green elements above the sequences represent α-helices, β-strands and coils in the structure of *X*. *campestris* GyrA CTD [38], respectively (PDB code: 3L6V chain A, as viewed by PyMOL [39]). Grey-filled triangle indicates the trypsinolysis site for EcGyrA (Arg571) [40] whereas unfilled triangles indicate those for PfGyrA (Arg854, Lys856 and Lys862). The putative coiled-coil of PfGyrA predicted by the SMART server is shown in red below the sequences in A. Annotations and smaller filled arrows underneath the sequences in A and B indicate the elements found in PfGyrA that are conserved with prokaryotic GyrA. **D.** X-ray crystal structure of *X*. *campestris* GyrA (PDB code: 3L6V [38]) shown in cartoon format with secondary structure elements coloured as described for A-C. Each of the six blades are numbered. The hydrophobic region of blade 1 is coloured orange and shown in stick format. The conserved S/T residue of blade 1 is shown in blue, in stick format and the conserved G of blade 1 is shown in cyan, in stick format.

Next, the amino acid sequence of PfGyrA was subjected to domain/motif prediction by Pfam [5] and SMART [27] domain prediction servers. As was previously reported [23], only three β-pinwheel blade motifs are predicted within PfGyrA-CTD by Pfam (Residue ranges: 978–1013, 1023–1068, and 1079–1121, Fig 1B), an unusual finding given that all known GyrAs contain six blades [7, 36, 37]. The lack of detection of motifs corresponding to blades 1, 2, and 6 of EcGyrA may be due to general sequence divergence, presence of *Plasmodium*-specific insertions, the absence of some of the defining features common to prokaryotic gyrases such as the GyrA-box, or simply the absence of the blade motif. In order to identify the conserved features of β-pinwheel motifs in PfGyrA, sequences of β-pinwheel motifs of type II topos whose structure has been determined were individually aligned (S3 Fig). Common features of β-pinwheel blade motifs in prokaryotic gyrases include a concentration of hydrophobic residues located in the first β-strand followed by the S/TxxG motif. An acidic residue is frequently found in the α-helix located on the N-terminus of the long loop that interfaces two blades. The long loop may contain a degenerate GyrA-box, and the Gly corresponding to the last Gly of the GyrA-box motif (QRRGGK**G**) is strongly conserved. One or two acidic residues are often found at the C-terminus of the loop between the second and the third β-strands. The third β-strand contains another region of hydrophobic residues. Several such features were found in PfGyrA-CTD in the regions corresponding to blade 1 and 2 of EcGyrA-CTD, but not for blade 6 (Fig 2). The putative blade 1 of PfGyrA-CTD has a hydrophobic region followed by a TxxG motif, and a Glu residue corresponding to EcGyrA-CTD Glu556 is present. A long *Plasmodium* specific insertion is found in a region corresponding to the loop region of blade motifs of prokaryotic GyrA. A short hydrophobic region is found at the C-terminus of the putative blade 1 of PfGyrA. A hydrophobic region and the strongly conserved glycine are found in the putative blade 2 of PfGyrA. No Ser or Thr directly corresponding to that of the S/TxxG motif is found, but Thr and Ser residues flanking the position corresponding to Ser594 of EcGyrA-CTD are frequently found in *Plasmodium* gyrases. These observations lead us to suggest the presence of putative β-pinwheel blades in PfGyrA-CTD, which were undetected by domain prediction servers. Accordingly, we hypothesise the presence of blades corresponding to 1–5 of EcGyrA-CTD in PfGyrA, but this does not exclude the possibility of a sixth blade, and further structural studies are necessary for verification. Accordingly this sequence was included in our truncated construct, consisting of residues 801–1222, which was cloned, expressed, purified, and subjected to biochemical analyses.

### Designing of constructs, expression & purification

Attempts were made to produce PfGyrA as truncated fragments, encompassing either the N-terminal or the C-terminal domain. Hereafter we focus in describing the production and analyses of the C-terminal domain (801–1222). PfGyrA-CTD with an N-terminal His-tag (His-PfGyrA-CTD) was successfully produced in soluble form using the cell-free protein synthesis system and purified to homogeneity. Cleavage of the N-terminal His-tag with TEV protease proved inefficient with the initial construct design. Therefore a Gly-Ser motif was inserted between the cleavage site and the PfGyrA-CTD and this led to an improvement in His-tag cleavage (S4 Fig). The resultant protein without the His-tag will be referred to as PfGyrA-CTD. Additionally, PfGyrA-CTD with a N-terminal MBP-tag was produced. This approach has enabled production of soluble PfGyrA-CTD in larger quantities. Protein produced by both approaches resulted in homogeneous samples as tested by analytical size exclusion chromatography (S5 Fig, calibration curve is given in S6 Fig). Molecular weights calculated from the elution volumes of both PfGyrA-CTD and MBP-PfGyrA-CTD indicate they, like EcGyrA-CTD (WT), are monomeric in solution (Table 1).

**Table 1:** Molecular weights of GyrA-CTD proteins calculated from their amino acid sequences and elution volumes from analytical size exclusion chromatography.

	Elution volume (ml)	Molecular weight, calculated from elution volume (kDa)	Molecular weight, calculated from amino acid sequence (kDa)
**MBP-PfGyrA-CTD**	1.65	109.6	89.7
**MBP-PfGyrA-CTD(ΔN)**	1.63	117.3	87.8
**PfGyrA-CTD**	1.95	39.4	49.6
**EcGyrA-CTD(WT)**	1.63	59.3	38.0

### Probing the folding of PfGyrA-CTD vs EcGyrA-CTD

**Fig 3:**
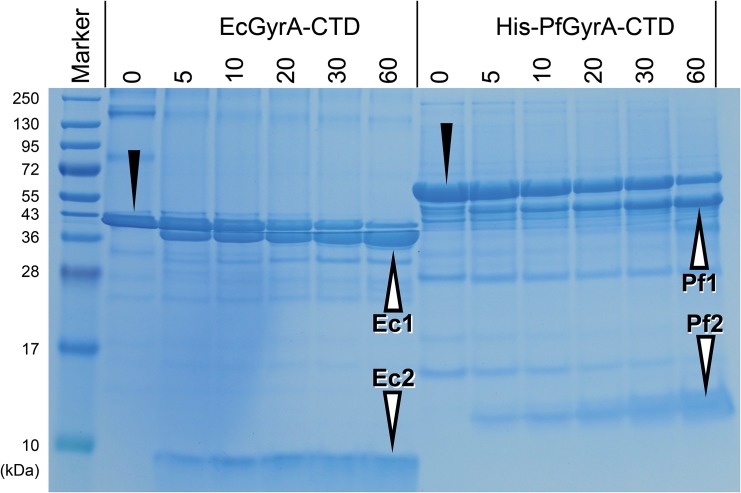
Limited trypsinolysis of PfGyrA-CTD. Protein digests were visualised as a function of time. Time elapsed in minutes after initiation of reaction is indicated along the top of the gel. Decreases in band intensities of the original proteins (black triangles) are associated with reciprocal increases in band intensities of the fragments (white triangles). Edman degradation has identified the following amino acid sequences from the denoted fragments. Ec1, 572-IKEED-576; Ec2 1-GSHME-2 [underlined Gly-Ser-His residues derived from pET28a(+)]; Pf1, 855-LKFND-859 (also 857-FNDLQ-861 and 863-GNEQE-867 at lower intensities); Pf2, MKDHL (derived from the N-terminal His-tag).

Limited proteolysis of *E*. *coli* GyrA has shown that the C-terminal peptide bond of Arg571, located C-terminal to the GyrA-box in the loop of blade 1, is prone to tryptic digestion [40]. To probe the global conformation of PfGyrA-CTD, the protein was subjected to limited trypsinolysis and aliquots taken at various time points were separated using SDS-PAGE. An overall similar digestion pattern was observed for both His-PfGyrA-CTD and EcGyrA-CTD(WT) (Fig 3). The undigested proteins at T = 0 appeared as a single band which was digested into a larger and a smaller fragment. Proteins from these bands were subjected to further analyses by N-terminal sequencing and MALDI-TOF. Both results confirm cleavage to have occurred at the C-terminal end of Arg854 in His-PfGyrA-CTD, and also at Lys856 and Lys862. All three cleavages sites are located within a *Plasmodium* specific insertion (Fig 2A). From the small decrease in the molecular weight of band Pf1 combined with the appearance of no band other than Pf2, it is likely that cleavage at the C-terminus, does not occur or is minimal. Such specific, limited cleavage sites suggest that His-PfGyrA-CTD assumes a well-folded globular structure. Secondly, conformational similarity to *E*. *coli* GyrA-CTD at more local level is implied by the similarity in digestion patterns, and this notion is supported by the sequence alignment which indicates the cleavage sites to occur between α1 and β3 of blade 1 for both His-PfGyrA-CTD and EcGyrA-CTD(WT).

In order to probe the secondary structure of PfGyrA-CTD, the CD-spectra between 190–350 nm were measured (S7 Fig). The CD-spectra and the calculated proportions (Table 2) indicate that His-PfGyrA-CTD is folded with an overall similar proportion of secondary structures as EcGyrA-CTD(WT).

**Table 2:** Proportion of secondary structures calculated from CD-spectra using the Dichroweb server [32].

	Helix	Strand	Turns	Unordered
**EcGyrA-CTD**	0.13	0.29	0.27	0.31
**PfGyrA-CTD**	0.13	0.31	0.24	0.33

### DNA binding properties of PfGyrA-CTD

The acidic C-terminal tail of EcGyrA was previously reported to inhibit the CTD from binding and introducing writhe into DNA [31], although the degree of this negative regulation is species dependent [41]. In order to investigate whether PfGyrA-CTD plays a similar role to bacterial gyrase CTDs, it was subjected to DNA-binding assays in the form of electrophoresis mobility shift assays (EMSA) and fluorescence anisotropy. Additionally, PfGyrA-CTD was subjected to a topology footprinting assay developed by Tretter and co-workers to test for its ability to wrap DNA [31]. Tests were carried out using PfGyrA-CTD without a His-tag and PfGyrA-CTD with a MBP tag. EcGyrA-CTD(ΔT) was employed as the positive control, since application of the topology footprinting assay and fluorescence anisotropy on EcGyrA-CTD(ΔT) has been demonstrated [31], and a similar CTD fragment of *X*. *campestris* GyrA has been shown to retard the migration of DNA in EMSA [38].

**Fig 4:**
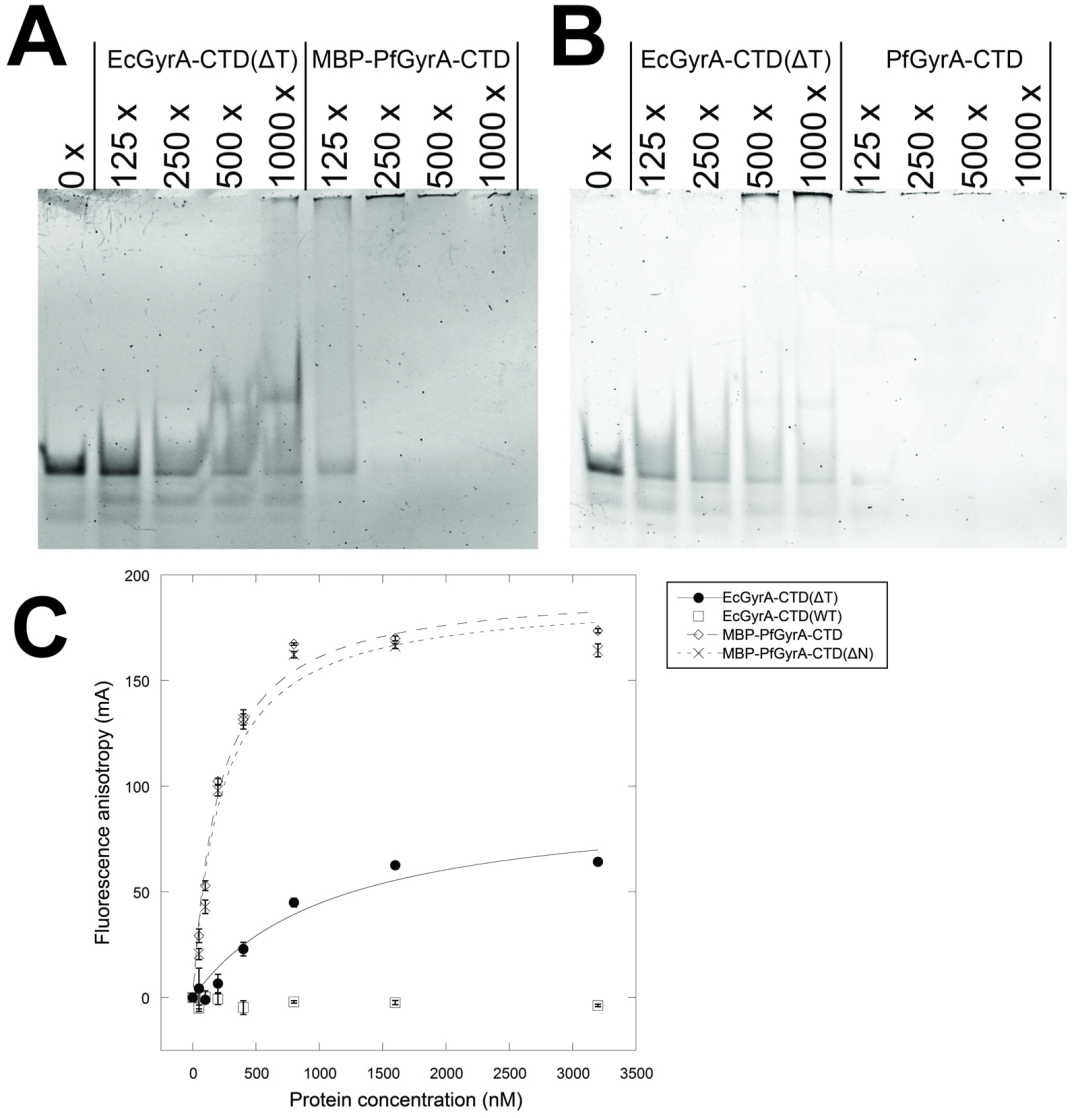
DNA binding by GyrA-CTD proteins demonstrated by EMSA and fluorescence anisotropy assays. **A** EcGyrA-CTD(ΔT) binds DNA as previously reported [34]. MBP-PfGyrA-CTD also binds DNA, apparently with higher affinity. **B** The experiment repeated with PfGyrA-CTD fragment without a tag. This CTD also exhibits a higher affinity for DNA. For A and B, protein concentration in terms of multiples of DNA concentration is given above each lane. **C** DNA binding by GyrA-CTD tested by fluorescence anisotropy. Fluorescence anisotropy by FAM-labelled DNA at 50 nM was measured as a function of protein concentration from 0 to 3200 nM. Error bars indicate standard deviations from three independent measurements. The presence and the absence of the acidic C-terminal tail (854–875) affects the DNA binding of EcGyrA-CTD negatively and positively, respectively, as previously reported [31] and were employed as control proteins in this assay. Bmax and Kd respectively for each protein are as follows. EcGyrA-CTD(ΔT), 94.28 mA ± 15.63 mA, 1114.60 nM ± 430.53 nM; MBP-PfGyrA-CTD, 194.32 mA ± 7.98 mA, 205.49 nM ± 31.40 nM; MBP-PfGyrA-CTD(ΔN), 189.82 mA ± 11.62 mA, 221.55 nM ± 49.50 nM. Values were not calculated for EcGyrA-CTD(WT).

EMSA of PfGyrA-CTD and MBP-PfGyrA-CTD revealed that both proteins have affinity for DNA (Fig 4). In both cases this appeared higher than that of EcGyrA-CTD(ΔT). Increasing the concentration of EcGyrA-CTD(ΔT) resulted in the disappearance of the free DNA band and upward streaking of the DNA indicating protein-DNA interaction. In the case of PfGyrA-CTD and MBP-PfGyrA-CTD, free DNA bound to protein at lower protein concentrations and was unable to enter the gel. In order to check if the disappearance of the DNA band from the gel is attributable to nuclease contamination, pUC19 plasmid was incubated with purified MBP-PfGyrA-CTD, and subjected to agarose gel electrophoresis. No major decreases in band intensities were observed when this incubation was carried out at 37°C for 4 hours with 3.2 μM of MBP-PfGyrA-CTD, ruling out digestion of substrate DNA by nuclease contamination (S8 Fig).

Fluorescence anisotropy was employed as an alternative method to probe protein-DNA interaction. MBP-PfGyrA-CTD was shown to bind FAM-labelled 37 bp dsDNA (Fig 4C). Higher signals for MBP-PfGyrA-CTD (89.7 kDa) compared to EcGyrA-CTD(ΔT) (35.6 kDa) may be explained by the larger molecular weight of the former. The calculated Kd of MBP-PfGyrA-CTD (0.21 μM ± 0.03 μM) was lower than that of EcGyrA-CTD(ΔT) (1.11 μM ± 0.43 μM), in agreement with the observations from EMSA. The Kd value for EcGyrA-CTD(ΔT) binding to DNA reported herein is higher than the previously reported value of 90 nM ± 21 nM [31], and this may be due to experimental variation between the two reports.

**Fig 5:**
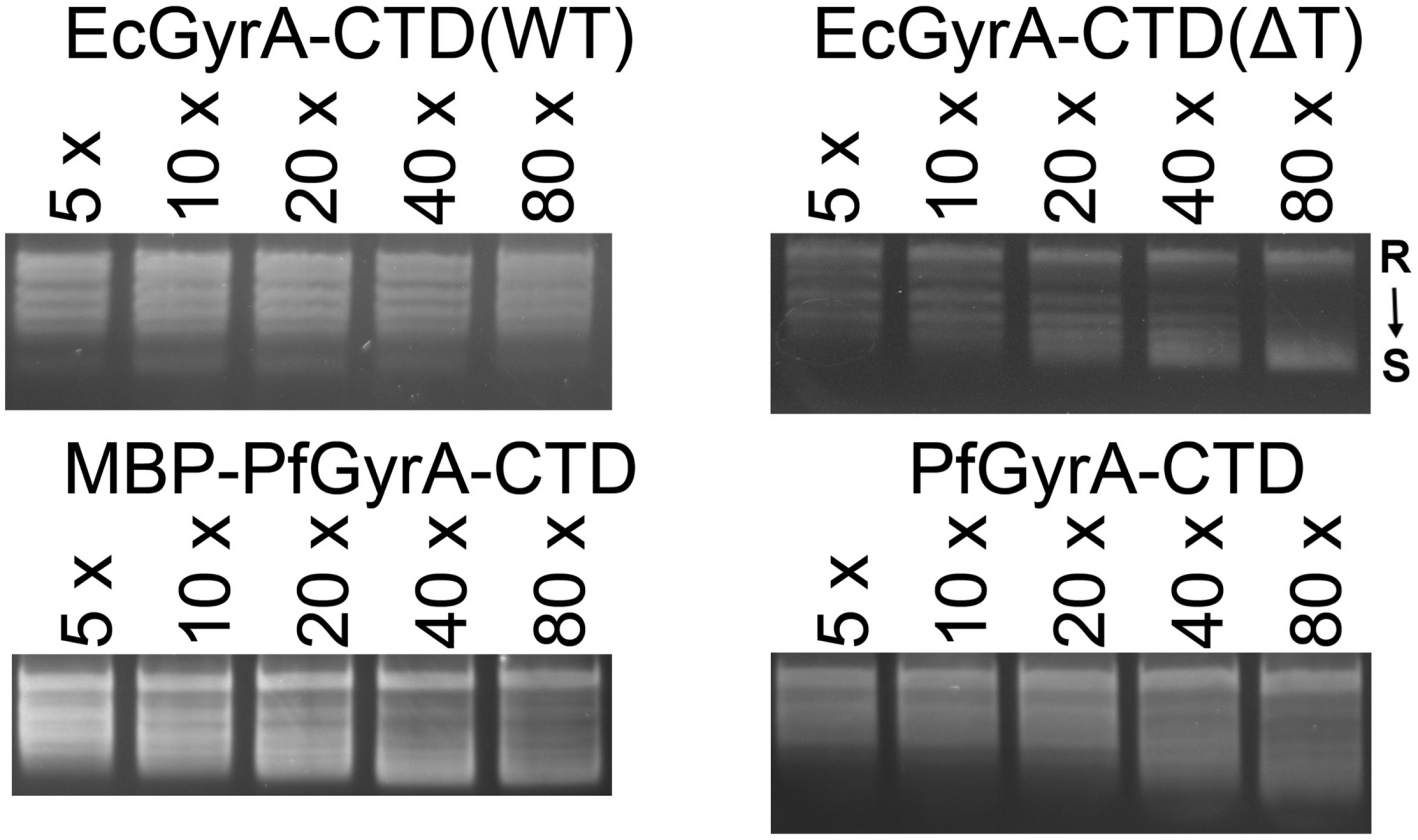
Topology footprinting assay for GyrA-CTD proteins. Ability of GyrA-CTD proteins to wrap DNA was visualised by incubating approx. 2.7 nM nicked (topologically free) DNA with the indicated molar excess of protein; subsequently the nick was sealed by T4 ligase, allowing the topological changes introduced as a result of protein-DNA interaction to be observed. EcGyrA-CTD(ΔT) and EcGyrA-CTD(WT) behaved as previously reported [31]. Similar to EcGyrA-CTD(ΔT), MBP-PfGyrA-CTD and PfGyrA-CTD introduced writhe into topologically free nicked plasmid DNA, and the effect increases in a concentration dependent manner. “R” and “S” represent the positions of relaxed and supercoiled DNA respectively and apply to all gels in the figure.

Having established the ability of PfGyrA-CTD to bind DNA, PfGyrA-CTD and MBP-PfGyrA-CTD were tested for their abilities to introduce writhe into DNA. As previously reported, the ability of EcGyrA-CTD(ΔT) to introduce writhe is manifested by the disappearance of the nicked plasmid band in response to increasing protein concentration, an effect not seen with EcGyrA-CTD(WT) (Fig 5). Similar to EcGyrA-CTD(ΔT), increasing concentrations of PfGyrA-CTD and MBP-PfGyrA-CTD had similar effects on DNA, albeit to a lesser extent. This indicates that PfGyrA-CTD can also wrap DNA, suggesting similarities between EcGyrA-CTD and PfGyrA-CTD in terms of their function, and by extension, their global conformations.

### Effect of deletion of Asn-rich region in PfGyrA-CTD

Having established that PfGyrA-CTD exhibits a similar phenotype to that of EcGyrA-CTD(ΔT), we next investigated the Asn-rich region in the putative blade 1 of PfGyrA-CTD (Fig 2A). Sequences of putative blade 1 of *Plasmodium* GyrA contain a *Plasmodium*-specific insertion in a region corresponding to the loop region of prokaryotic GyrA-CTD blade 1. *P*. *falciparum* gyrase proteins (both GyrA and GyrB) are unique among *Plasmodium* spp. in that not only do they feature a quantitatively higher proportion of Asn residues, but they are qualitatively unique for their Asn-rich regions [23]. The *P*. *falciparum* proteome is rich in such Asn-repeats, and this has led to several hypotheses concerning their functionality [42]. Here, to probe the role of the repeat, MBP-PfGyrA-CTD without the Asn-rich region (887-QNNNNDNNNNNNDNNN-902) was produced, and the behaviour of the mutant was tested.

**Fig 6:**
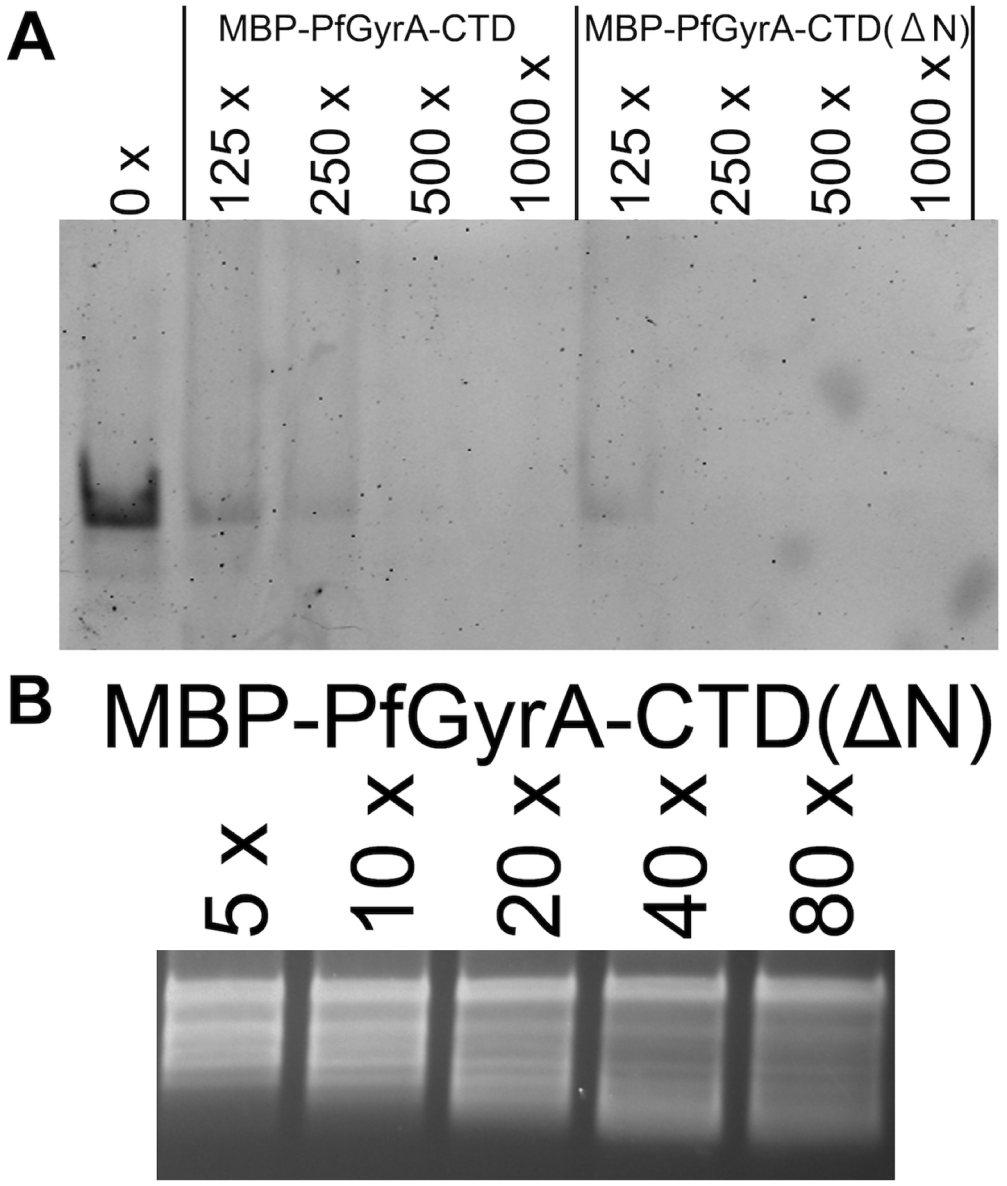
Analyses of the MBP-PfGyrA-CTD variant without the Asn-rich region. MBP-PfGyrA-CTD without the Asn-rich region (887–902) was subjected to **A.** EMSA in the presence of 1 nM substrate 140 bp DNA and the indicated excess of protein and **B**. topology footprinting assay performed as in Fig 5. MBP-PfGyrA-CTD(ΔN) exhibits similar affinity for DNA when compared to MBP-PfGyrA-CTD. No difference could be observed in terms of ability to wrap DNA.

The MBP-PfGyrA-CTD without the Asn-rich region [denoted as MBP-PfGyrA-CTD(ΔN)] was purified, and subjected to EMSA and topology footprinting assays (Fig 6) and fluorescence anisotropy (Fig 4C). In all cases, the lack of the Asn-rich region made no obvious difference, indicating that it does not affect the functionality of the protein as far as the present tests are concerned. Analytical size exclusion chromatography had already indicated MBP-PfGyrA-CTD and PfGyrA-CTD to be monomeric in solution, and this was unchanged with MBP-PfGyrA-CTD(ΔN) (S5 Fig). This indicated that, at least in context of the present construct design, the Asn-rich region in PfGyrA-CTD does not cause inter-molecular aggregation. In light of these data, it may be that the Asn-rich region does not form a well-folded structural element, but rather an extensive coil-region as expected from its single-amino acid rich nature. The close proximity between the trypsinolysis sites and the Asn-rich region in the present study supports this notion. If the Asn-rich region is not involved in core aspects of the protein function, then its removal may result in protein that is more amenable to crystallisation (as the proportion of disordered region is inversely correlated with the probability of protein crystallisation [43]).

## Discussion

In the present study, a region corresponding to the C-terminal domain of prokaryotic GyrA protein was predicted in GyrA of *P*. *falciparum*. We were able to produce this protein by several methods but had difficulties in the production of soluble PfGyrA proteins in full length forms. This may be due to the lack of cognate molecular chaperone such as Hsp110 [44] together with the presence of an Asn-rich region in the putative blade 1 of PfGyrA-CTD (residue range 887–902, Fig 1), which could potentially be detrimental to the protein stability as has been previously discussed [42].

Overall the results achieved with the C-terminal domain of prokaryotic GyrA indicate that residues 801–1222 are well-folded as judged by CD-spectroscopy and limited proteolysis. Additionally, limited proteolysis suggested the presence of a flexible loop in PfGyrA in the region corresponding to the first blade of EcGyrA-CTD. This result is consistent with the sequence alignment, and thus legitimises the prediction of a blade motif not detected by domain prediction servers. MBP-PfGyrA-CTD was shown to bind to DNA with a Kd of approximately 200 nM, which is lower than that of EcGyrA-CTD(ΔT) with a Kd of approximately 1100 nM. In EMSA experiments, the protein-DNA complexes for PfGyrA-CTD and MBP-PfGyrA-CTD do not enter the polyacrylamide gel at all (Fig 4). This could be due to non-specific aggregation effects but is more likely due to the higher calculated isoelectric points of PfGyrA-CTD (9.68) and MBP-PfGyrA-CTD (9.26) compared to EcGyrA-CTD(ΔT) (5.66). Additionally, the higher pI values of PfGyrA-CTD and PfGyrA-CTD may offer an explanation for their apparently higher affinities towards DNA. It is noteworthy that the absorption spectrum of MBP-PfGyrA-CTD immediately after affinity purification indicated the presence of DNA (S9 Fig), thus necessitating the use of a high-salt buffer to isolate the protein from DNA. This may be another indication of the potent DNA binding activity by PfGyrA-CTD. Similarly to EcGyrA-CTD(ΔT), MBP-PfGyrA-CTD and PfGyrA-CTD were able to introduce writhe into DNA, and this may suggest similarity in the overall protein structure as well as charge distribution between these two proteins.

Prediction servers predicted three β-pinwheel blades between 978–1121 of PfGyrA, and a coiled-coil between 829–859, the latter being in close agreement to that proposed previously [34]. These predictions can be regarded with some confidence. However, a sequence from an evolutionary divergent protein may result in false negatives. Given the experimental data presented in this study, we propose PfGyrA-CTD to possess at least five β-pinwheel blade motifs within its CTD. The C-terminal region where no blade motif is predicted is expected to be well-folded as judged by the lack of outstanding cleavage following limited proteolysis. This is despite many potential cleavage sites in this region (18 Lys and 1 Arg between residues 1122–1222 of PfGyrA). Additionally, some secondary structures can be predicted in the C-terminus (S10 Fig). The exact structure of this region, along with the number of blade motifs awaits verification by structure determination.

We have provided significant empirical data supporting the prediction of a CTD at residues 801–1222. Previously the CTD was predicted between 1022–1121 (100 residues) [35], along with a coiled-coil region between 824–855 (contained in a construct termed “PfGyrACC”, residues 723–887). In contrast, the EcGyrA-CTD (531–875) consists of 345 residues and contains six β-pinwheel motifs [36]. A number of type II topos with less than six β-pinwheel motifs are known, and it is possible that PfGyrA-CTD may be such a domain. However, given that our proposed PfGyrA-CTD (422 residues) exhibited DNA wrapping activity, it seems likely that it presents a more complete functional domain. The role of PfGyrA-CTD remains to be elucidated but DNA binding and wrapping results suggests it includes a similar role to the CTD of prokaryotic gyrase complexes. Also, PfGyrA-CTD was found to be monomeric, regardless of the presence of an MBP-tag. This contrasts with previous work suggesting that the C-terminal region of PfGyrA forms a dimer via the putative coiled-coil [35]. The present data do not conclusively exclude the presence of the putative coiled-coil nor the fact that when present in a shorter construct it may have effects such as dimerization not seen in longer proteins.

Although appearing to have significant differences compared to bacterial GyrA CTDs, the role of the domain in *P*. *falciparum* remains unclear. Unlike prokaryotic cells that possess topo IV and gyrase that act antagonistically, PfGyrase is to date the only known type II topo in the apicoplast [24] (an apicomplexan eukaryotic topoisomerase II external to the apicoplast is known and has recently been purified and investigated [45]). The existence of PfGyrase raises the possibility that it may possess a dual functionality like *M*. *tuberculosis* gyrase. Recent data on another apicoplast gyrase from *T*. *gondii* supports this idea, showing that enzyme to have dual supercoiling and decatenation activities [46]. In the case of *M*. *tuberculosis* gyrase, activity was shown to be modulated by calcium ions that bind to the putative EF-hand motif between the NTD and the CTD [47]. PfGyrA is noted for its particularly long *Plasmodium* specific insertion between the N-terminal and the C-terminal domains (Fig 1B). An analogous mechanism to that of *M*. *tuberculosis* gyrase may be present via such a *Plasmodium* specific insert, acting to modulate the activity of PfGyrase between gyrase-like and Topo IV-like activities.

## Supporting Information

S1 Fig Preparation of relaxed plasmid DNA. Substrate for topology footprinting assay was prepared by introducing a nick in pUC19 plasmid using Nb.BbvCI nicking endonuclease. Lane one shows the untreated pUC19 plasmid material, lane 2 shows the relaxed nicked product.
(TIFF)

S2 Fig Difficulties in producing full-length PfGyrA proteins. Arrows indicate the protein of interest where applicable. Total (i.e. both soluble and insoluble fractions) and soluble fractions were subjected to SDS-PAGE following cell-free syntheses using various PfGyrA constructs. CTD fragment was soluble, but full-length PfGyrA (163–1222) became mostly insoluble regardless of the type of the N-terminal tag (His only, His+TrxA (where Trx = thioredoxin), or His+SUMO) or the presence of the chaperones (DnaK, DnaJ and GrpE). +C and−C indicate the presence and the absence of the chaperones during the synthesis reaction, respectively. T = Total fractions of the protein synthesis reaction. S = Soluble fraction of the protein synthesis reaction.
(TIF)

S3 Fig Sequence alignment of structurally characterised blade motifs of C-terminal domains of type II topoisomerases. Secondary structures of blade 1 of *X*. *campestris* GyrA (PDB code: 3L6V) are shown above the aligned sequences (Red, α-helix; yellow, β-strand; green, coil). Only strongly conserved secondary structure elements are numbered (from the N-terminus). Residues/regions that are conserved among blade motifs are indicated below the aligned sequences. The abbreviated species name and their PDB codes of the CTD structures are as follows (all are of gyrase, unless otherwise specified): Aa, *Aquifex aeolicus*, 3NO0; Bb, *Borrelia burgdorferi*, 1WP5; Ec, *Escherichia coli*, 1ZI0; Gs, *Bacillus stearothermophilus*, 1SUU; Mt, *Mycobacterium tuberculosis*, 3CU1 and 4G3N; Xc, *Xanthomonas campestris*, 3L6V, EcParC, ParC of *Escherichia coli*, 1ZI0. The numbers following the abbreviated species names indicate the blade number within the C-terminal domain.
(TIF)

S4 Fig Efficient His-tag cleavage by TEV protease. Removal of the His-tag from the expressed PfGyrA-CTD was only possible after inserting the Gly-Ser motif between the TEV cleavage site and the PfGyrA-CTD. Application of TEV protease to proteins containing the Gly-Ser motif (His-GS-PfGyrA-CTD) results in decreases in molecular weights, but no decrease was found in the protein without the Gly-Ser motif (His-PfGyrA-CTD) following TEV-treatment. “Chaperone” refers to the presence (+) or absence (-) of the chaperones (DnaK, DnaJ and GrpE) in the expressing cells. “Before” refers to before treatment with TEV.
(TIF)

S5 Fig Size exclusion chromatography of GyrA-CTD proteins. Proteins were subjected to isocratic elution, using buffer containing 300 mM NaCl, 20 mM Tris/HCl, pH 8.0. For MBP fusion proteins, buffers were supplemented with 5 mM maltose in order to minimise the non-specific interaction between MBP and the resin matrix [30]. Comparison between the molecular weights calculated using elution volumes and amino acid sequences indicate that the tested proteins are likely to be monomers in solution.
(TIF)

S6 Fig Calibration of the analytical size exclusion chromatography column. **A** Combined chromatograms. Chromatograms were normalized by areas under curves. **B** Calibration curve was created using Excel 2010 (Microsoft). Y is the elution volume in ml, and x is the log_10_ of the molecular weight (Da) of the standard proteins. Superdex 200 5/150 GL (GE Healthcare) was calibrated using the following standards; Bovine serum albumin (BSA, Sigma), hen egg white lysozyme (HEWL, Wako), blue dextran (GE Healthcare), aldolase (GE Healthcare), and ferritin (GE Healthcare).
(TIF)

S7 Fig CD-spectra of GyrA-CTD proteins. Spectra were collected for EcGyrA-CTD (dark grey diamonds) and PfGyrA (light grey squares).
(TIF)

S8 Fig Testing MBP-PfGyrA-CTD for the presence of nuclease contamination. 300 ng of pUC19 plasmid was incubated with 3.2 μM MBP-PfGyrA-CTD in a buffer (70 mM KCl, 20 mM Tris/HCl, 10% Glycerol, 1 mM MgCl_2_, pH 7.5) at 37°C for 4 hours. No visible decrease in DNA band intensities were observed, therefore contaminating nuclease was deemed absent from the MBP-PfGyrA-CTD sample. Presence of buffers and/or the protein changed the migration patterns of plasmids, however. Lane 1, DNA in deionised water; Lane 2, DNA in buffer; Lane 3, DNA in buffer with 3.2 μM MBP-PfGyrA-CTD.
(TIF)

S9 Fig DNA is co-purified with MBP-PfGyrA-CTD during affinity purification. SDS-PAGE analysis (**A**) and absorption spectrum (**B**) of the MBP-PfGyrA-CTD immediately after affinity purification. Following size exclusion chromatography with a high-salt buffer (**C**), the protein of interest was collected from the peak at 73.45 ml (indicated with a black filled arrow) deemed to contain a smaller proportion of DNA as judged by a lower A260/A280 ratio (**D**) than the bulk sample after the affinity purification.
(TIF)

S10 Fig Secondary structure prediction of PfGyrA. **A** Secondary structure prediction of full-length PfGyrA is shown along with the (B) schematic domain diagram of the protein. Expended view of the secondary structure prediction of the C-terminus of PfGyrA is also shown (C). Colour scheme for the domain diagram is the same as in Fig 1. No conserved residues were found to support the presence of a β-pinwheel blade motif in the region corresponding to the 6^th^ blade of prokaryotic GyrA in PfGyrA (Fig 2C). Nonetheless this region in PfGyrA is predicted to be folded, because some secondary structures are predicted by the SOPMA server (see S1 References) and little evidence of tryptic cleavage at the C-terminus is seen in limited proteolysis (Fig 3).
(TIF)

S1 References (DOCX)
