# Supplementary figures and images for: Investigating the Roles of the C-Terminal Domain of Plasmodium falciparum GyrA
Source: PLoS One. 2015 Nov 13;10(11):e0142313. doi: 10.1371/journal.pone.0142313 (PMC4643928; doi:10.1371/journal.pone.0142313)

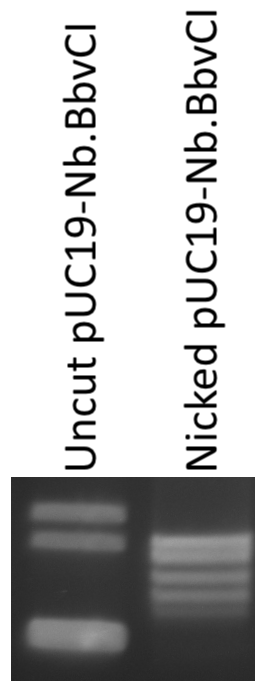

Supplement: S1 Fig — Substrate for topology footprinting assay was prepared by introducing a nick in pUC19 plasmid using Nb.BbvCI nicking endonuclease. Lane one shows the untreated pUC19 plasmid material, lane 2 shows the relaxed nicked product. (TIFF) [file pone.0142313.s001.tiff]

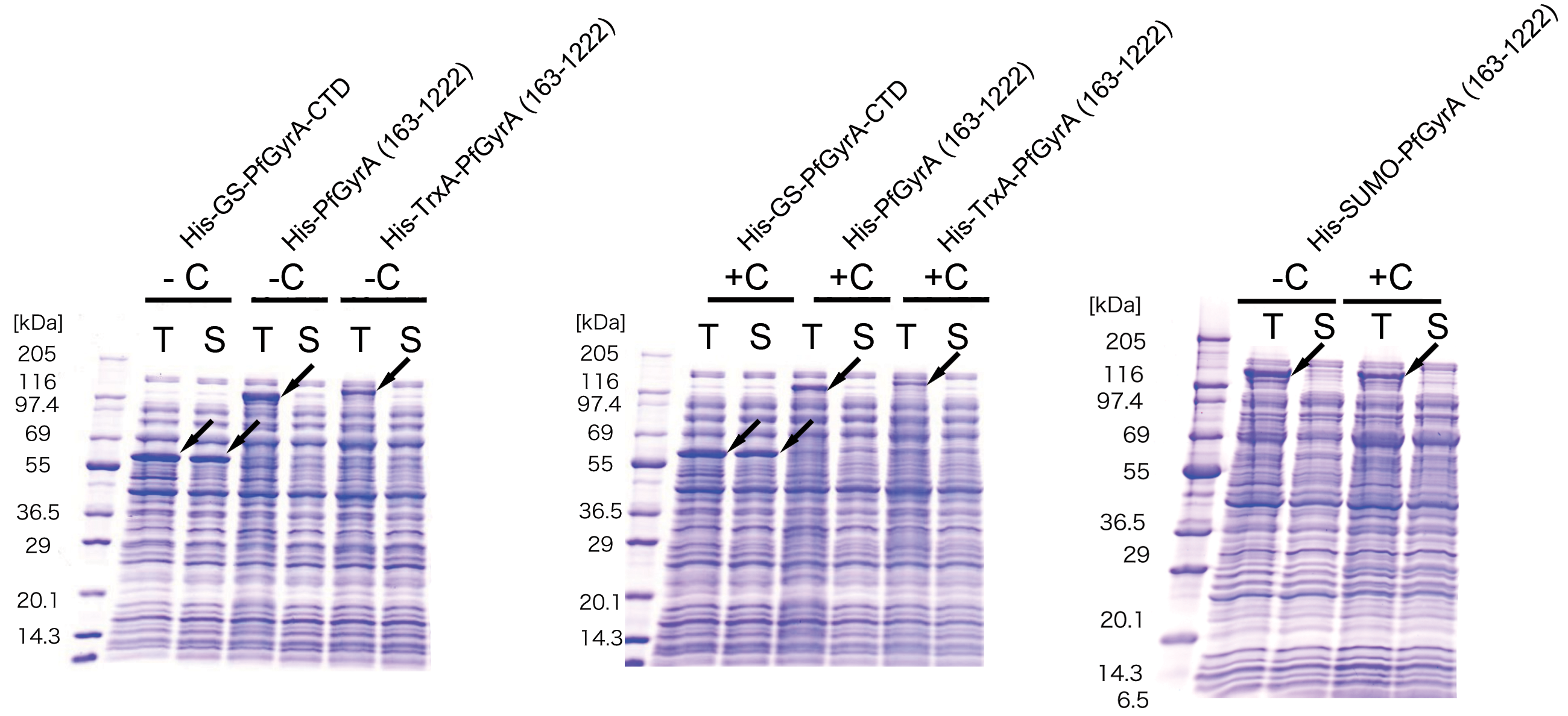

Supplement: S2 Fig — Arrows indicate the protein of interest where applicable. Total (i.e. both soluble and insoluble fractions) and soluble fractions were subjected to SDS-PAGE following cell-free syntheses using various PfGyrA constructs. CTD fragment was soluble, but full-length PfGyrA (163–1222) became mostly insoluble regardless of the type of the N-terminal tag (His only, His+TrxA (where Trx = thioredoxin), or His+SUMO) or the presence of the chaperones (DnaK, DnaJ and GrpE). +C and−C indicate the presence and the absence of the chaperones during the synthesis reaction, respectively. T = Total fractions of the protein synthesis reaction. S = Soluble fraction of the protein synthesis reaction. (TIF) [file pone.0142313.s002.tif]

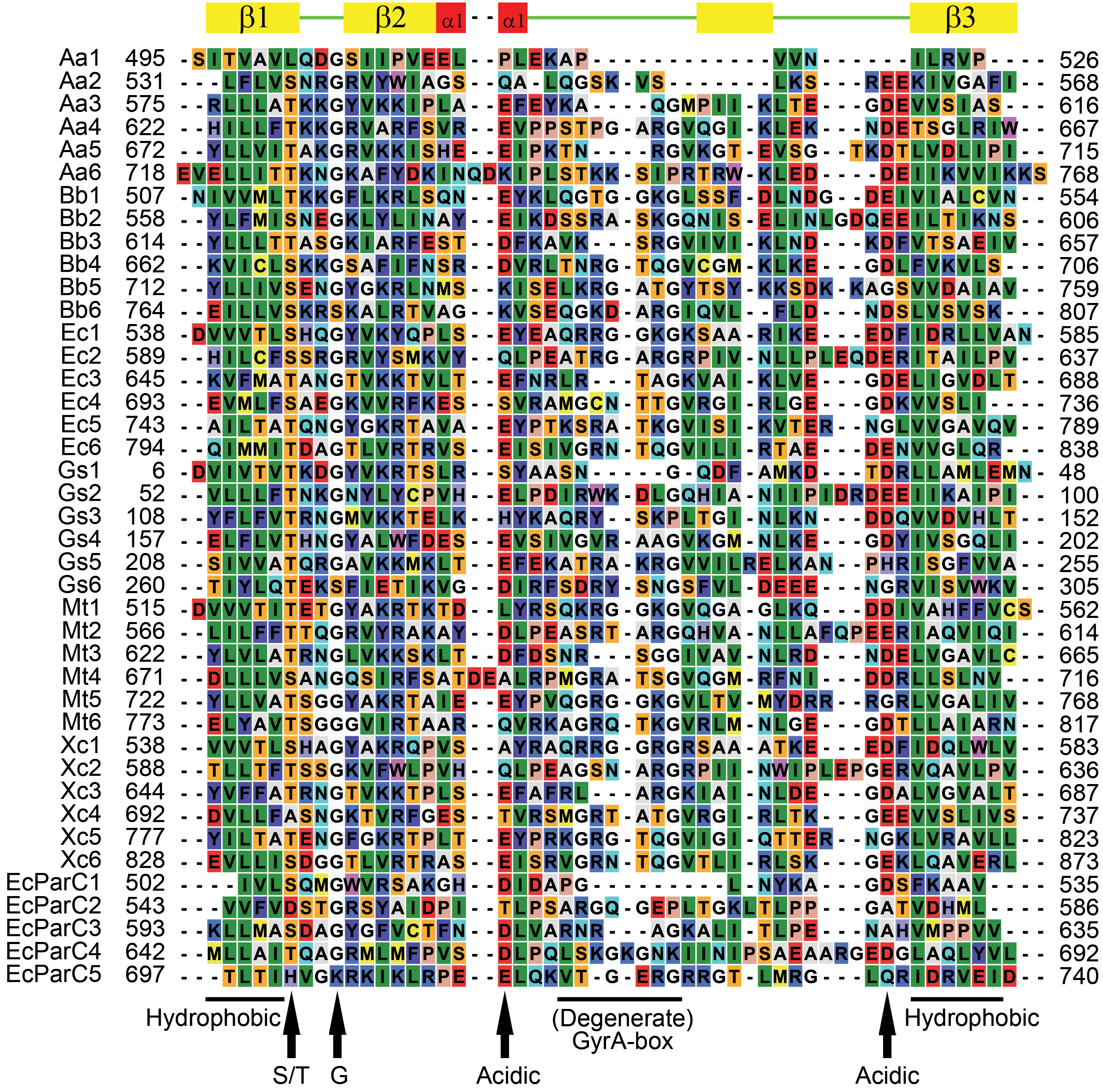

Supplement: S3 Fig — Secondary structures of blade 1 of X. campestris GyrA (PDB code: 3L6V) are shown above the aligned sequences (Red, α-helix; yellow, β-strand; green, coil). Only strongly conserved secondary structure elements are numbered (from the N-terminus). Residues/regions that are conserved among blade motifs are indicated below the aligned sequences. The abbreviated species name and their PDB codes of the CTD structures are as follows (all are of gyrase, unless otherwise specified): Aa, Aquifex aeolicus, 3NO0; Bb, Borrelia burgdorferi, 1WP5; Ec, Escherichia coli, 1ZI0; Gs, Bacillus stearothermophilus, 1SUU; Mt, Mycobacterium tuberculosis, 3CU1 and 4G3N; Xc, Xanthomonas campestris, 3L6V, EcParC, ParC of Escherichia coli, 1ZI0. The numbers following the abbreviated species names indicate the blade number within the C-terminal domain. (TIF) [file pone.0142313.s003.tif]

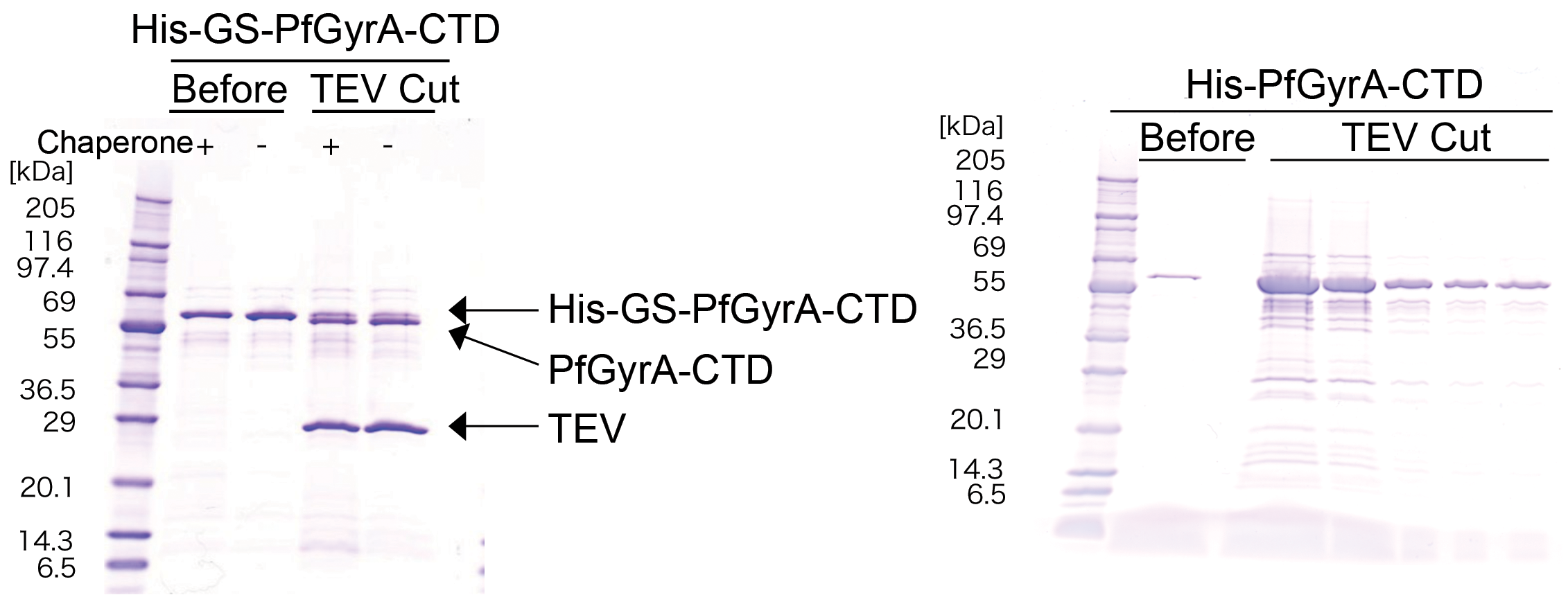

Supplement: S4 Fig — Removal of the His-tag from the expressed PfGyrA-CTD was only possible after inserting the Gly-Ser motif between the TEV cleavage site and the PfGyrA-CTD. Application of TEV protease to proteins containing the Gly-Ser motif (His-GS-PfGyrA-CTD) results in decreases in molecular weights, but no decrease was found in the protein without the Gly-Ser motif (His-PfGyrA-CTD) following TEV-treatment. “Chaperone” refers to the presence (+) or absence (-) of the chaperones (DnaK, DnaJ and GrpE) in the expressing cells. “Before” refers to before treatment with TEV. (TIF) [file pone.0142313.s004.tif]

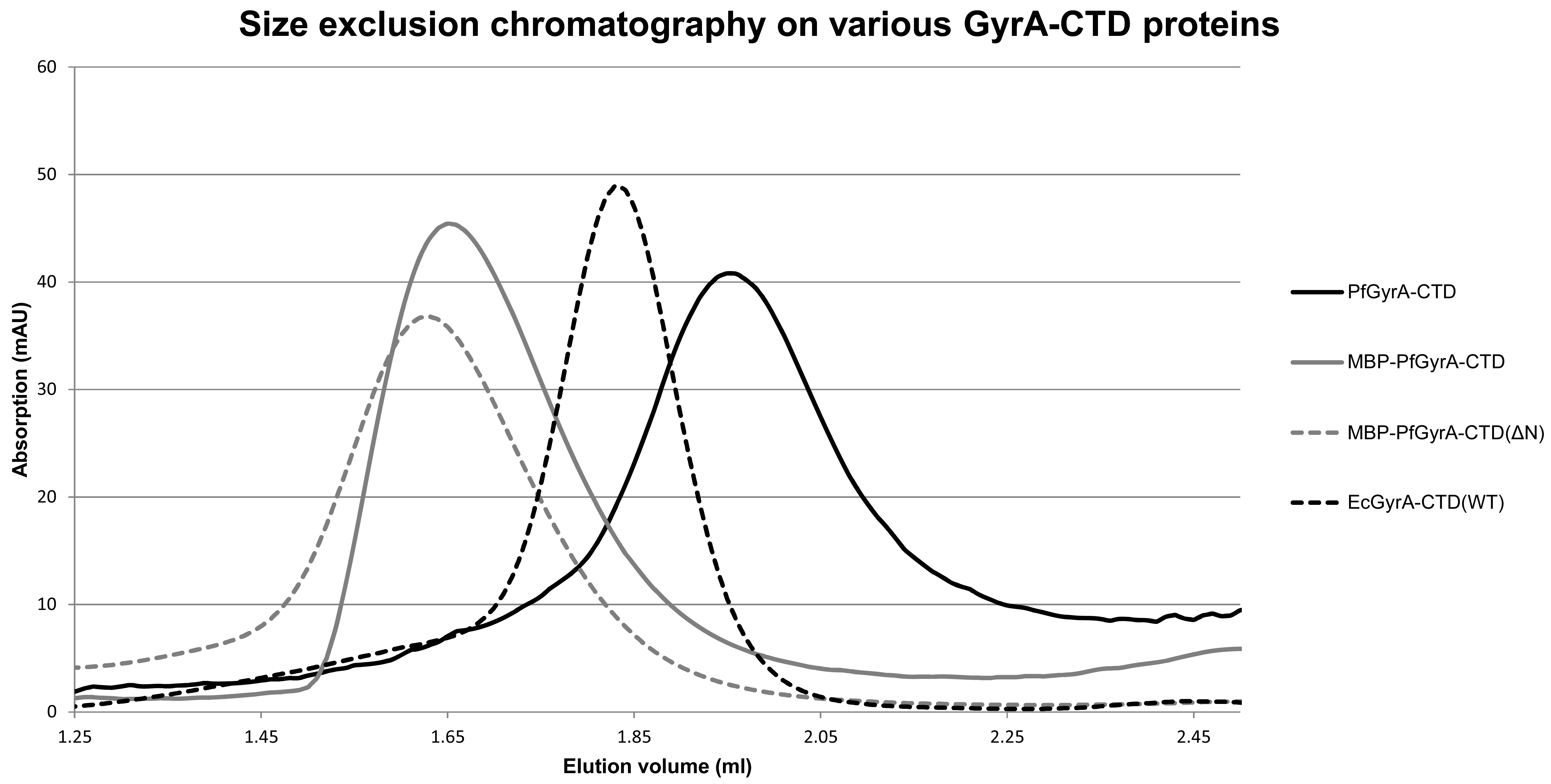

Supplement: S5 Fig — Proteins were subjected to isocratic elution, using buffer containing 300 mM NaCl, 20 mM Tris/HCl, pH 8.0. For MBP fusion proteins, buffers were supplemented with 5 mM maltose in order to minimise the non-specific interaction between MBP and the resin matrix [30]. Comparison between the molecular weights calculated using elution volumes and amino acid sequences indicate that the tested proteins are likely to be monomers in solution. (TIF) [file pone.0142313.s005.tif]

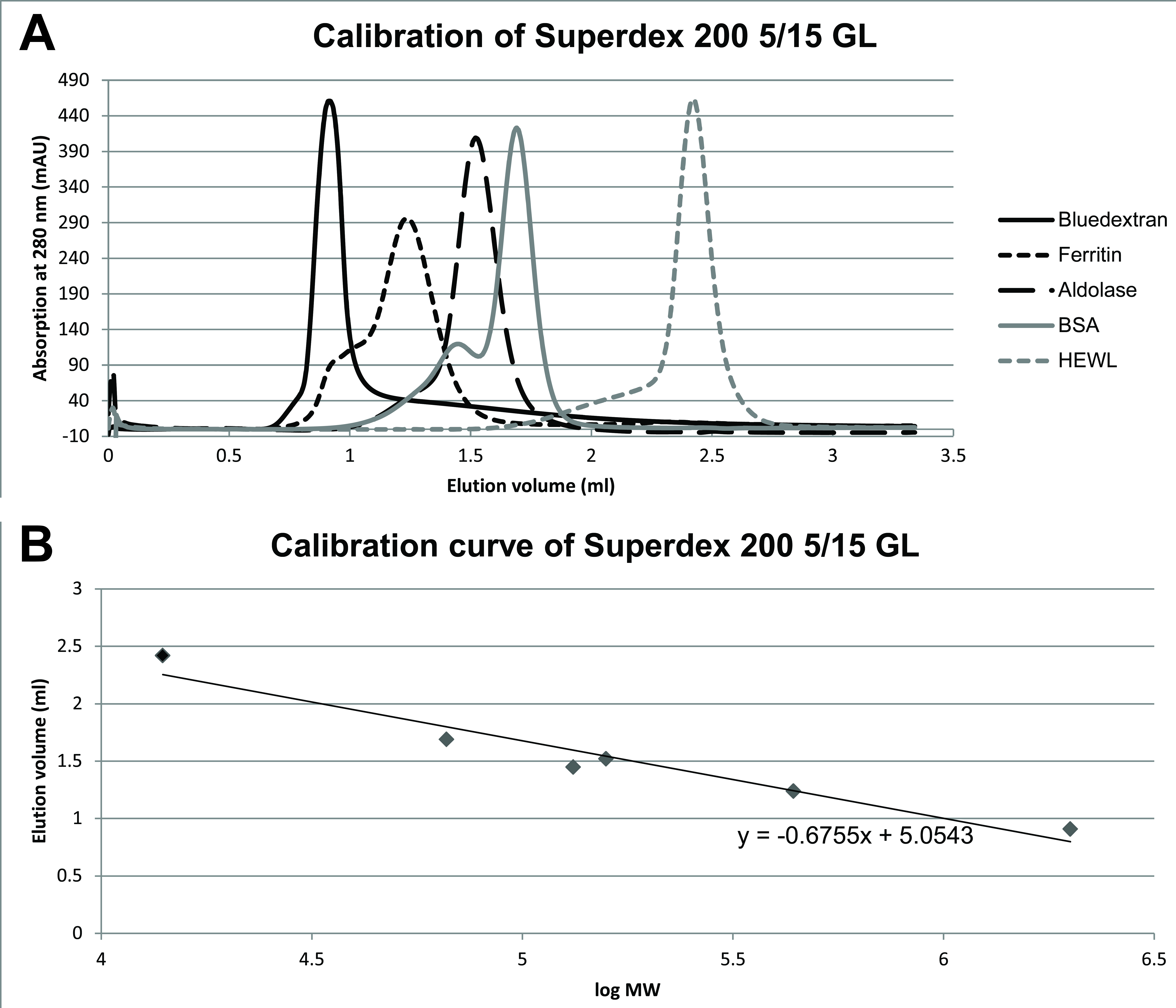

Supplement: S6 Fig — A Combined chromatograms. Chromatograms were normalized by areas under curves. B Calibration curve was created using Excel 2010 (Microsoft). Y is the elution volume in ml, and x is the log10 of the molecular weight (Da) of the standard proteins. Superdex 200 5/150 GL (GE Healthcare) was calibrated using the following standards; Bovine serum albumin (BSA, Sigma), hen egg white lysozyme (HEWL, Wako), blue dextran (GE Healthcare), aldolase (GE Healthcare), and ferritin (GE Healthcare). (TIF) [file pone.0142313.s006.tif]

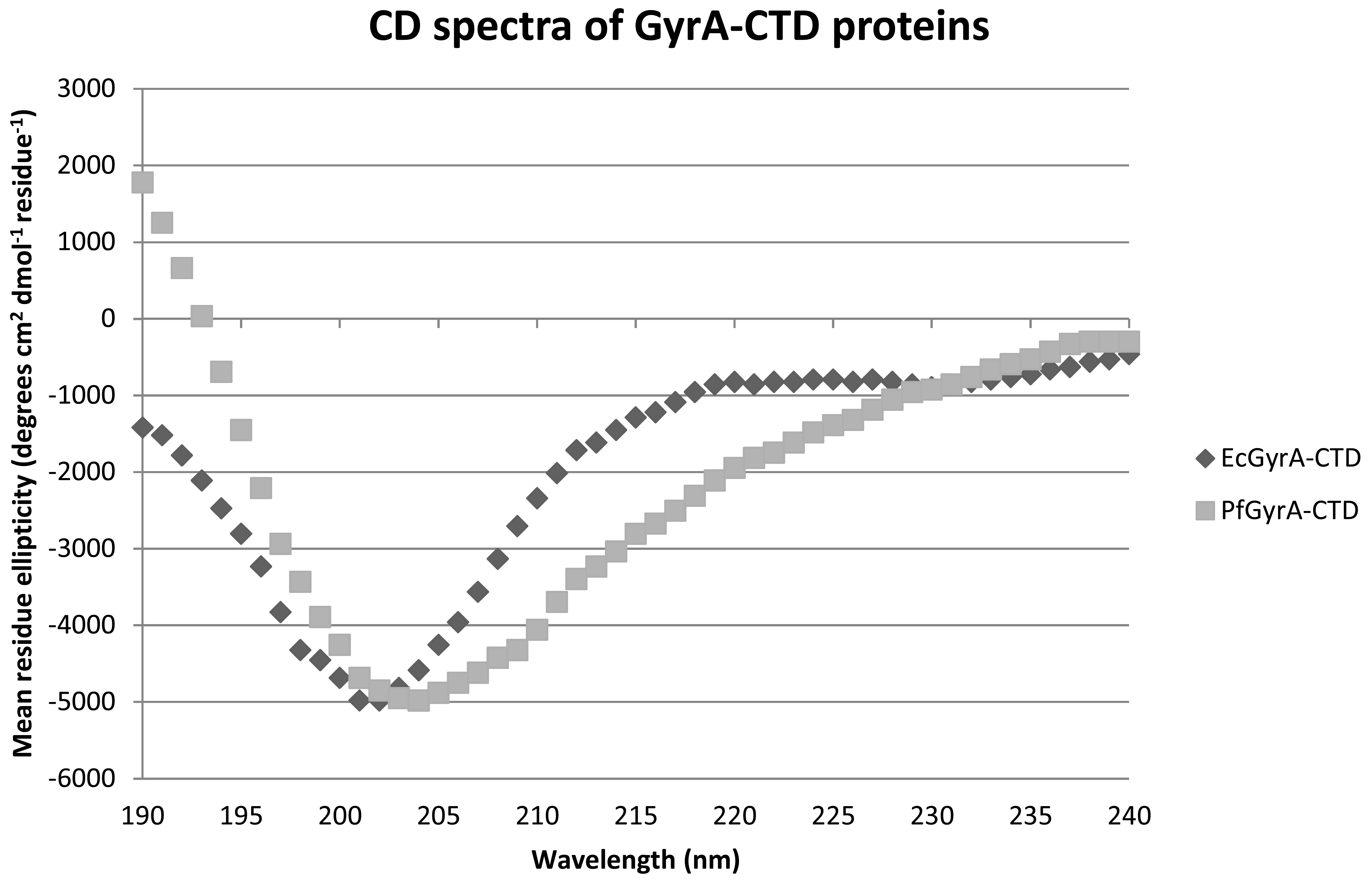

Supplement: S7 Fig — Spectra were collected for EcGyrA-CTD (dark grey diamonds) and PfGyrA (light grey squares). (TIF) [file pone.0142313.s007.tif]

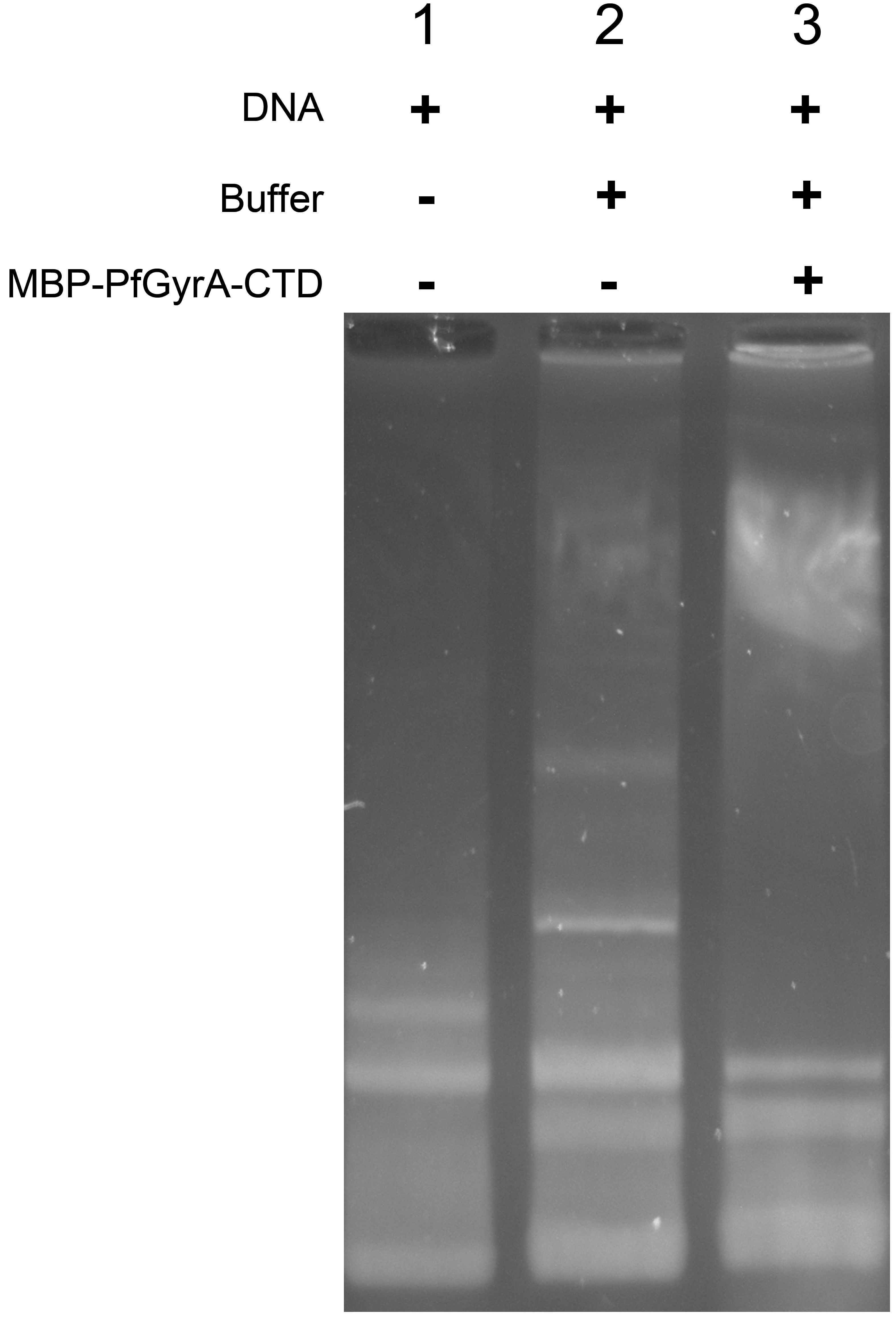

Supplement: S8 Fig — 300 ng of pUC19 plasmid was incubated with 3.2 μM MBP-PfGyrA-CTD in a buffer (70 mM KCl, 20 mM Tris/HCl, 10% Glycerol, 1 mM MgCl2, pH 7.5) at 37°C for 4 hours. No visible decrease in DNA band intensities were observed, therefore contaminating nuclease was deemed absent from the MBP-PfGyrA-CTD sample. Presence of buffers and/or the protein changed the migration patterns of plasmids, however. Lane 1, DNA in deionised water; Lane 2, DNA in buffer; Lane 3, DNA in buffer with 3.2 μM MBP-PfGyrA-CTD. (TIF) [file pone.0142313.s008.tif]

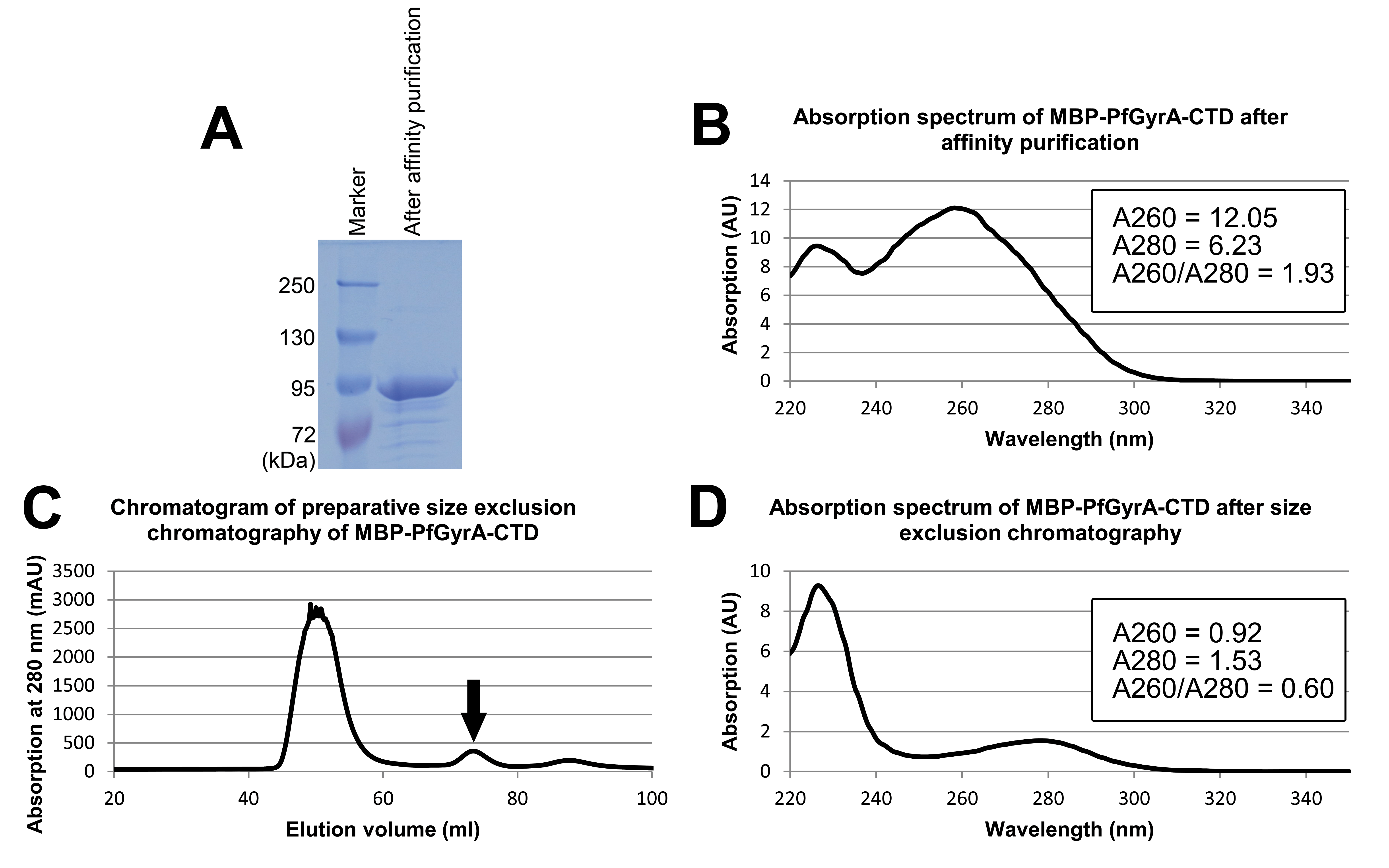

Supplement: S9 Fig — SDS-PAGE analysis (A) and absorption spectrum (B) of the MBP-PfGyrA-CTD immediately after affinity purification. Following size exclusion chromatography with a high-salt buffer (C), the protein of interest was collected from the peak at 73.45 ml (indicated with a black filled arrow) deemed to contain a smaller proportion of DNA as judged by a lower A260/A280 ratio (D) than the bulk sample after the affinity purification. (TIF) [file pone.0142313.s009.tif]

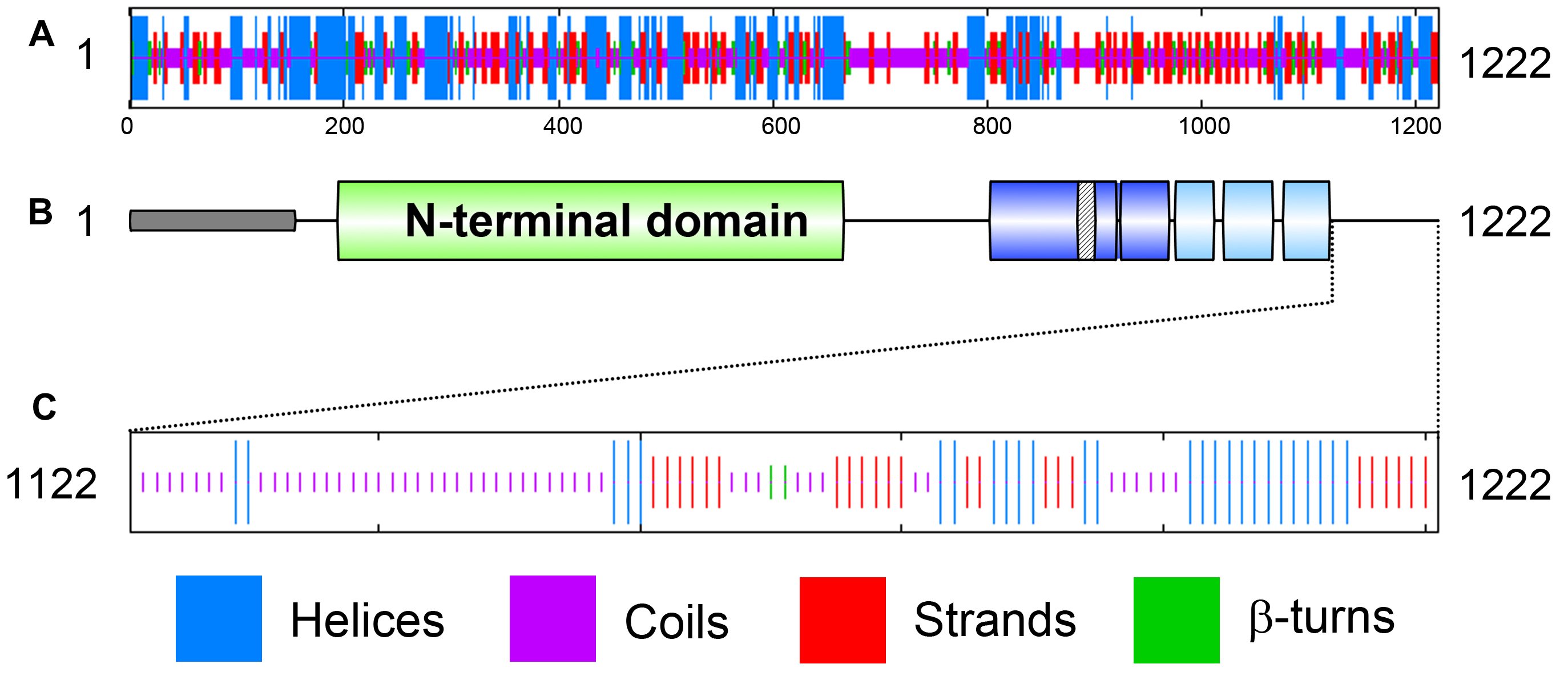

Supplement: S10 Fig — A Secondary structure prediction of full-length PfGyrA is shown along with the (B) schematic domain diagram of the protein. Expended view of the secondary structure prediction of the C-terminus of PfGyrA is also shown (C). Colour scheme for the domain diagram is the same as in Fig 1. No conserved residues were found to support the presence of a β-pinwheel blade motif in the region corresponding to the 6th blade of prokaryotic GyrA in PfGyrA (Fig 2C). Nonetheless this region in PfGyrA is predicted to be folded, because some secondary structures are predicted by the SOPMA server (see S1 References) and little evidence of tryptic cleavage at the C-terminus is seen in limited proteolysis (Fig 3). (TIF) [file pone.0142313.s010.tif]
