# Supplementary material for: Investigating the Roles of the C-Terminal Domain of Plasmodium falciparum GyrA
Source: PLoS One. 2015 Nov 13;10(11):e0142313. doi: 10.1371/journal.pone.0142313 (PMC4643928; doi:10.1371/journal.pone.0142313)
Supplement: S1 References — (DOCX) [file pone.0142313.s011.docx]

# S1 References

# 1. Geourjon C, Deleage G (1995) SOPMA: significant improvements in protein secondary structure prediction by consensus prediction from multiple alignments. Comput Appl Biosci 11: 681-4.
